# Evaluation and Molecular Characterization of Colistin-Resistant Isolates of *Pseudomonas aeruginosa* and *Klebsiella pneumoniae* from the Infected Wounds of Hospitalized Patients

**DOI:** 10.4014/jmb.2407.07005

**Published:** 2024-08-16

**Authors:** Munaza Ijaz, Madiha Khan, Haya Yasin

**Affiliations:** 1 Department of Microbiology, University of Central Punjab, Lahore 54000, Pakistan; 2 Department of Pharmaceutical Sciences, College of Pharmacy and Health Sciences, Ajman University, Ajman 346, United Arab Emirates

**Keywords:** Antibiotic resistance, antibacterial susceptibility, colistin, PCR, resistance gene

## Abstract

This study was planned to determine the colistin-resistant (CR) gene distribution among two species of gram-negative bacteria, *Pseudomonas aeruginosa* and *Klebsiella pneumoniae*. In total, 50 isolates of *K. pneumoniae* (14 isolates, 28%) and *P. aeruginosa* (36 isolates, 72%) were isolated between August 2023 and October 2023 from clinical wound samples at Jinnah Hospital and Lahore General Hospital Lahore, Pakistan. To determine the resistance genes linked to CR and assess antimicrobial susceptibility, all isolates were kept at -80°C in 15% glycerol broth. Using the right primer sets, a polymerase chain reaction (PCR) was utilized to identify the CR-associated *mcr-1* gene of the gram-negative isolates. Out of 50, 40 isolates (80%) showed resistance against colistin with MICs of 8 and 128 μg/ml. The majority (97%) of *P. aeruginosa* CR strains were considered multidrug resistant (MDR). All *K. pneumoniae* isolates were resistant to cefepime, cotrimoxazole, ceftriaxone, and imipenem. The clinical CR isolates of *P. aeruginosa* were highly resistant to ceftriaxone, imipenem, cefepime, cotrimoxazole, ciprofloxacin, amikacin, and piperacillin/tazobactum. The antibiotic resistance pattern was terrifyingly high among both bacterial species. According to the PCR results, CR was prevalent among the gram-negative samples, and the *mcr-1* gene was positive in 6/40 (15%) of the CR isolates, including four *P. aeruginosa* and two *K. pneumoniae* strains. The high CR (80%) reported in this research is cause for concern and underscores an urgent need to use colistin in a limited and logical manner, similar to other antibiotics.

## Introduction

Antimicrobial resistance refers to the ineffectiveness of antimicrobials [1]. This is a significant issue since a resistant illness has the potential to cause death and may be transmitted to others at substantial cost to people and society [2]. Researchers have been working for years to improve data comparability and enhance understanding of highly drug-resistant bacteria, particularly those for which there are no effective treatment options available.

Polymyxins, a collection of polypeptide antibiotics comprising five different chemical entities (polymyxins A-E), were first identified in 1947 [4]. Polymyxin B and polymyxin E (colistin) are the two antibiotics that have been primarily used in clinical settings [4]. Polymyxins exert activity against certain gram-negative bacteria, including *Pseudomonas aeruginosa*, and are being used worldwide for topical treatments [5].

Polymyxin E, also known as colistin, is a cationic polypeptide. This antibiotic kills gram-negative bacteria by targeting the lipopolysaccharide molecules of the cell membrane to exert its pharmacological effect [6]. In the past, genetic mutations in the organism’s chromosomes were considered responsible for colistin resistance (CR). These mutations arise at considerable cost to the organism’s overall fitness and cannot be transferred to other organisms. Nonetheless, there are various drawbacks of colistin as an antibiotic moiety. For example, it produces neurotoxicity and nephrotoxicity. Despite these undesired effects, colistin remains one of the last effective therapeutic choices for invasive bloodstream infections due to carbapenemase-producing gram-negative rods [7].

In recent years, excessive use of colistin has resulted in the development of resistance to this antibiotic [8] in several bacterial species, including *Vibrio cholera* and *Serratia marcescens* [9]. Moreover, recent studies have revealed the extensive prevalence of CR isolates that have gained their resistance through chromosomal genes or plasmids [10]. Meanwhile, other studies have linked CR to a mutation in a two-component regulatory system governed by chromosomal genes [11].

The gene *mcr-1* is a member of the phosphoethanolamine transferase enzyme family. Chinese researchers first identified the plasmid-mediated CR gene in 2015 in *Escherichia coli* (*E. coli*) strains isolated from human, animal, and food sources [12]. Since then, other Enterobacteriaceae strains possessing the *mcr-1* gene have also been identified worldwide [13]. Following the gene’s discovery, further studies showed many variations of *mcr-1*, such as *mcr-2* and *mcr-3* [14]. The emergence of this CR gene may hinder the treatment of Enterobacteriaceae infections while stimulating efforts to develop new protocols for healthcare professionals and laboratory testing [15]. Gram-negative bacteria can survive with or without oxygen and are recognized by their potential to degrade glucose and produce nitrate and catalase enzymes [16]. These bacteria cause nosocomial infections such as pneumonia and are gaining increasing resistance to a large number of currently available antibiotics. Moreover, such pathogens have characteristic features that need exploring as new sources of resistance, especially since they may transfer genetic moieties that facilitate other bacteria in inducing resistance to these drugs.

The CDC has developed guidelines aimed at adopting practical methods to stop the spread of MDR and extensively drug resistant (XDR) gram-negative bacteria. *P. aeruginosa* and *E. coli* are responsible for most of the nosocomial gram-negative infections [17]. Such infections have become rampant and represent a significant danger to public health. In addition to being difficult to treat, they also have high rates of morbidity and mortality. Consequently, colistin is being utilized afresh as the last resort for treatment due to the increasing resistance of gram-negative bacteria to the existing antibiotics.

In this study, we aimed to uncover and analyze the distribution of CR genes among gram-negative bacteria isolated from clinical wound samples collected at Jinnah Hospital and Lahore General Hospital Lahore, Pakistan.

## Material and Methods

Selective agars such as mannitol salt agar (Sigma-Aldrich, USA), cetrimide agar (HiMedia Laboratories, India), and MacConkey agar (HiMedia Laboratories) were purchased from various companies. Mueller-Hinton Agar (Thermo Fisher Scientific, UK), nutrient agar, and LB agar from Sigma-Aldrich was purchased through local vendors, Antibiotic disks were purchased from Scientific Laboratory, Lahore, Pakistan. The reverse and forward primers were purchased from SYNBIO Technologies, Italy. A Thermo Scientific Gene JET Plasmid Miniprep Kit was used for plasmid extractions, and the Bio-Rad T100 Thermal Cycler (Singapore) was used for polymerase chain reaction (PCR).

### Clinical Sample Collection

Following a basic cleaning of the wounds using normal saline, samples were obtained from 50 patients (*n* = 50) of various age groups and genders. This was done by using a sterile swab (Clinix, Lahore) that had been moistened beforehand. The swab was rotated across a 1 cm^2^ area of the wound surface in a zig-zag motion, starting from the center and moving towards the outer edges of the wound. Next, the swab was inserted into the tube containing the transport medium (Clinix). Each sample was obtained prior to applying an antibiotic to the wound using a sterile cotton swab, to prevent any contamination from the normal microorganisms present on the skin.

### Bacterial Strains

Fifty gram-negative strains (14 *K. pneumoniae*, and 36 *P. aeruginosa*) were isolated from different clinical wound specimens (burn, surgical and diabetic) from Jinnah Hospital and Lahore General Hospital, Lahore, Pakistan, from August to October of 2023.

### Sample Processing

To determine the presence of CR gram-negative bacteria, all clinical wound samples underwent bacteriological processing in accordance with the appropriate recommendations [1]. The samples were appropriately marked with the patient's ID number, lab ID number, date, time, and mode of collection. They were then transported to the laboratory in an insulated cold box with ice packs, in accordance with the recommendations set by the World Health Organization (WHO) [2]. However, samples that were not well marked, inadequately transported with evident indications of contamination, and lacking full patient information, were not included.

### Standard Microbiological Testing

All clinical samples were processed using standard microbiological procedures, which included culture, microscopy, biochemical testing, and observation of colonial characteristics of bacteria on selected culture medium. The analysis was performed by streaking the samples on blood agar and nutrient agar plates. The identification was done by observing the typical features of colonies on selective medium. The colonies were transferred onto nutrient agar from the Gram stain slides in the incubator, where the temperature was set at 37°C for 24 h, which is shown in normal medium. The isolates from cetrimide selective agar and MacConkey agar were verified as *K. pneumoniae* and *P. aeruginosa* using bacteriological and microscopic examinations. In addition to this, there were other routine identification methods performed, including the catalase oxidase citrate indole pigmentation urease procedure [7]. Similarly, with a 15% glycerol solution at -80°C, the *K. pneumoniae* and *P. aeruginosa* isolates were obtained and preserved [8].

### Antibiotic Sensitivity Testing

The Kirby Bauer disc diffusion technique was used to test for antibiotic susceptibility in all isolates on Mueller-Hinton agar using antibiotics recommended by the Clinical and Laboratory Standards Institute (CLSI) 2020 guidelines. The antibiotics used in the study were amikacin (AK, 30 μg), ciprofloxacin (CIP, 5 μg), imipenem (IPM, 10 μg), piperacillin-tazobactum (TZP, 110 μg), colistin (CT, 10 μg), cefepime (FEP, 30), cotrimoxazole (SXT, 25 μg), and ceftriaxone (CRO, 30 μg). Antibiotic resistance was reported in three groups or more idepressant categories and the isolates therein are classified as multiple-drug resistant strains (MDRS) [15].

### Testing of Minimal Inhibitory Concentration (MIC)

We assessed colistin resistance in this study using a broth microdilution technique incorporating colistin sulfate powder (Sigma Aldrich) and 96-well, round-bottom microtiter plates [12]. To make the bacterial suspension, four to six colonies from bacterial growths were inoculated into cation-adjusted Mueller Hinton Broth (Sigma Aldrich). The bacterial suspension was calibrated to a 0.5 McFarland standard, which corresponded to a bacterial concentration of 1 - 2 × 10^8^ CFU/ml. The bacterial solution was diluted at a ratio of 1:20 to get a final consultation of bacterial growth of 1 × 10^8^ CFU/ml. A stock solution of colistin sulfate was produced using sterile distilled water. In addition, colistin was diluted in a microtiter plate well using a 2-fold serial dilution method. The dilutions ranged from 0.25 μg/ml to 128 μg/ml and were prepared in 100 μl of sterile CAMHB solution. A bacterial solution of 50 μl was added to each well, except the negative control. The positive control was generated by adding a bacterial suspension with the highest concentration. The lowest concentration of MIC [14] did not result in any growth. The microtiter plates were placed in an incubator and kept at 37°C for 24 h. Following incubation, the growth in each well was quantified using a microplate reader configured to measure the optical density (OD) at the specific wavelength of 600 nm. The MIC data were analyzed based on the CLSI guidelines [14]. The forthcoming MIC values on the microplate reader were calculated by estimating the growth inhibition rate (GIR) using the following method:

$$GIR=\frac{V_{o}-V1}{V_{o}}\times 100\%.$$

The GIR is expressed as a percentage. The OD of the control group is denoted as V_o_, whereas the OD of the test group is denoted as V_1_ [6].

### Preparation of Bacterial Cultures and Extraction of *mcr-1* Plasmids from CR Strains

The investigation was done on *mcr-1*-carrying strains of *P. aeruginosa* and *K. pneumoniae*. The strains were isolated using biosafety techniques, maintained by the addition of 15% glycerol and stored at a temperature of -80°C. Plasmids were removed using the Thermo Scientific GeneJET Plasmid Miniprep Kit, following the manufacturer's procedure. To recap, 10 ml of bacteria-inoculated Luria-Bertani broths were quantitatively transferred into 15 ml centrifuge tubes (Sigma-Aldrich). The tubes were centrifuged at 10,000 ×*g* for 5 min at 4°C while the medium bacteria were then re-suspended in RB buffer (Promega) using 250 μl Ringer's solution and later placed into 1.5 ml microcentrifuge tubes. Then, 250 μl of LB buffer was added into the microcentrifuge tube and rotated to mix it completely before it was left in the incubator for 5 min. Lastly, 350 μl of NB buffer was added, and then inverted 4 to 6 times to be agitated immediately. Later, the mixture obtained was spun at 10,000 ×*g* (×*g*=1000 rcf where rcf=relative centrifugal force) for 10 min at 4°C. We then moved the aqueous fraction to a tiny column via pipette, and spun it at 10,000 ×*g* for 2 min at 4°C. This mini-column was eluted and re-aspirated into a collecting tube. To that, 700 μl of WB buffer was added and then centrifuged at 10,000 ×*g* for 1 min at 4°C. The resulting mixture was then moved to a new receiver tube. Afterwards, a new 1.5 ml microtube was utilized by the mini-column. Then, 50 μl of EB Buffer was added, following a 10 min incubation period at ambient temperature, following which it was centrifuged at 10,000 ×*g* for 1 min while being chilled at 4°C. In the end, the retrieved plasmids were kept for further investigation at a temperature below -80°C. The material was subjected to gel electrophoresis on a 1.2% agarose gel to confirm the plasmid extraction [10].

The plasmids were examined for the presence of the *mcr-1* gene using a PCR test. The *mcr-1* gene has an amplicon size of 309 [20]. The PCR was conducted using a 12-μl reaction mixture consisting of the following components: 7.5 μl of PCR Master Mix reagent from Thermo Scientific, 1.5 μl of *mcr-1*-F primer (5 -CGGTCAGTCCGTTTGTTC-3) and *mcr-1*-R primer (5 -CTTGGTCGGTCTGTAGGG-3), 2 μl of DNA template, and 7.5 μl of nuclease-free water. The performance of the PCR involved the following different cycling parameters: a preliminary denaturation step at 94°C for 5 min, followed by 25 cycles consisting of denaturation at 94°C for 30 s, annealing at 57°C for 30 s, and extension at 72°C for 50 s. The final extension was carried out at a temperature of 72°C for 5 min. Following the PCR, the DNA amplicons were detected by using agarose gel electrophoresis with a 1.5% agarose gel [11].

### Statistical Analysis

Findings were compared through Student’s *t*-tests. The level of significance was set at 0.05. SPSS version 20.0 was used to conduct statistical analysis.

## Results

### Phenotypic Analysis

On blood agar, *P. aeruginosa* colonies had a smooth, mucoid, greenish appearance with beta hemolysis and emitted a grape-like odor. In addition, they exhibited pigment synthesis, were oxidase-positive, and showed motility. On the other hand, *K. pneumoniae* appeared as mucoid, slimy, pink colonies on MacConkey agar, non-hemolytic, string test positive, oxidase negative and non-motile.

### Distribution of *P. aeruginosa* and *K. pneumoniae* among Different Body Sites

**Fig. 1:**
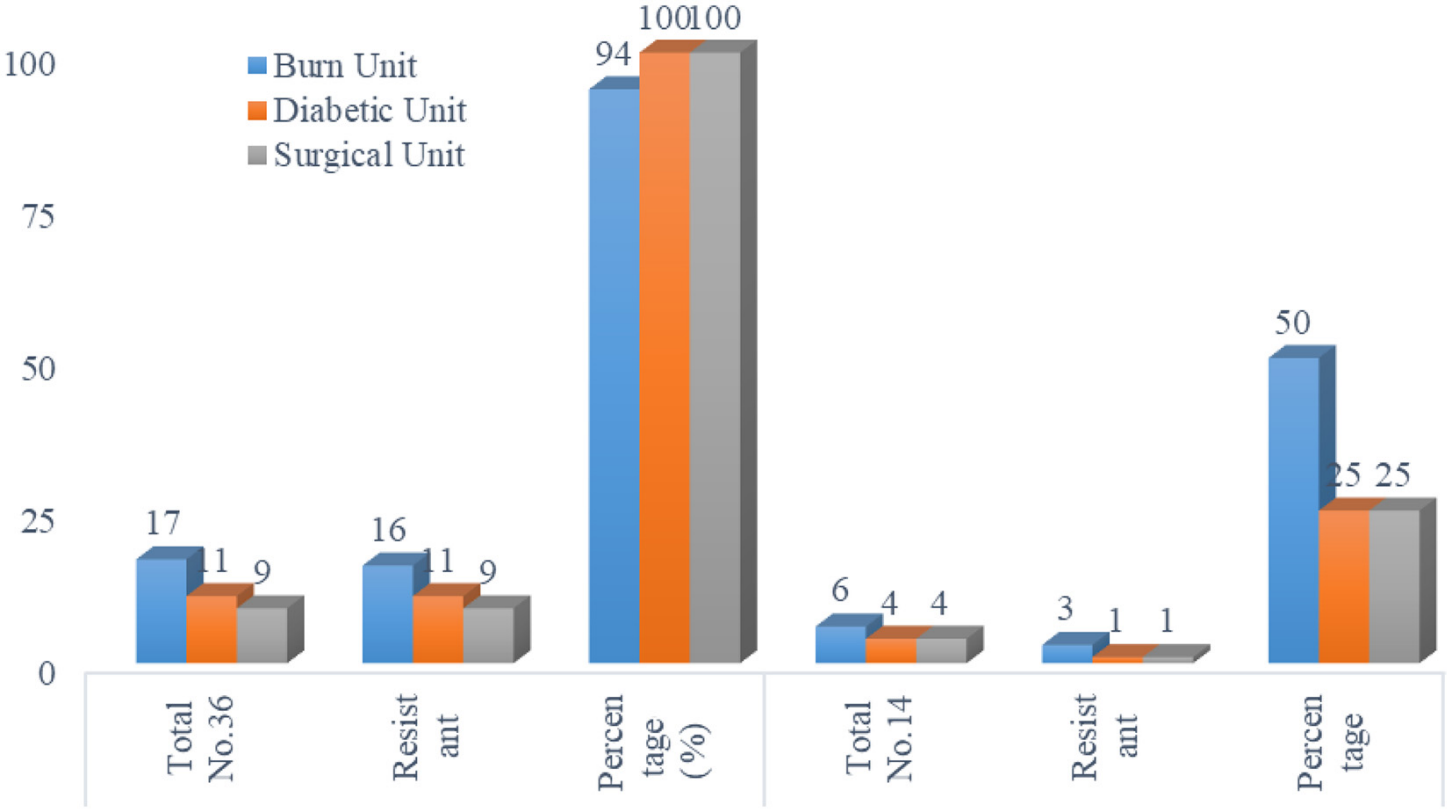
Distribution of *P. aeruginosa* and *K. pneumoniae* among different body sites, with most of the CR strains being recovered from the burn unit (46%), and the lowest rate of strains recovered from the surgical unit (24%), while the diabetic unit showed a 30% rate.

A total of 50 isolates of *P. aeruginosa* (36) and *K. pneumoniae* [14] were obtained from the collected wound specimens. Most of the CR strains were recovered from the burn unit (46%), and the lowest rate of strains was recovered from the surgical unit (24%), while the diabetic unit showed a 30% rate (Fig. 1).

### Demographic Data

The average age of the patients included in the research was 43.1 years. The largest number (22) of strains were obtained from patients aged 20 to 40, whilst the smallest number (6) of strains were obtained from individuals aged 40 to 60. Out of 50 isolates, 40 isolates (35 isolates of *P. aeruginosa* and 5 isolates of *K. pneumoniae*) were found to be CR. Data suggested that patients aged between 20-40 years showed the highest percentage (44%), with 30%in the 0–20 year-old group, 14% in the 60–80 group, and 12% of patients aged between 40-60 years. Among those studied, 66% of patients were male, while 34% were female (*p* < 0.05).

### Susceptibility Testing

Among the collected wound samples, 50 gram-negative bacterial strains were obtained as follows: *P. aeruginosa* (36 isolates, 72%) and *K. pneumoniae* (14 isolates, 28%). Out of 50 isolates, 40 bacteria (80%) were observed to be CR (Table 1). All five CR isolates of *K. pneumoniae* were resistant to cefepime, cotrimoxazole, ceftriaxone, and imipenem. The clinical CR isolates of *P. aeruginosa* resistant to ceftriaxone, imipenem, cefepime and cotrimoxazole were 35, 34, 34, and 33, respectively. In this study, *K. pneumoniae* and *P. aeruginosa* isolates showed significant (*p* < 0.05) antibiotic-resistance patterns, as the majority of the isolates showed resistance against most of the antibiotics. Out of 50 isolates, 40 (80%) were resistant to colistin. The CR isolates of *K. pneumoniae* showed 100%resistance to carbapenem, while 97% of *P. aeruginosa* isolates were resistant to carbapenem.

### Testing of MIC

**Fig. 2:**
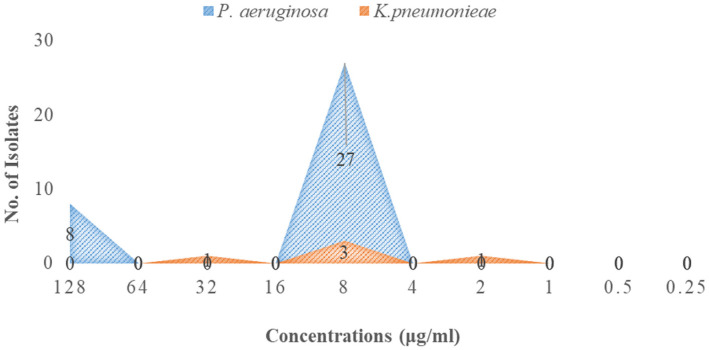
MIC values of the CR strains of *P. aeruginosa* and *K. pneumoniae*. Among the 35 *P. aeruginosa* isolates tested in this study, the majority (27 isolates) exhibited an MIC = 8 μg/ml. However, 8 isolates of *P. aeruginosa* showed a higher MIC of 128 μg/ml. For *K. pneumoniae*, out of the 5 isolates examined, 3 isolates displayed an MIC of 8 μg/ml. One *K. pneumoniae* isolate demonstrated a higher MIC of 32 μg/ml, while another isolate showed a lower MIC of 2 μg/ml.

In this study, we analyzed the antibiotic susceptibility profiles of *P. aeruginosa* and *K. pneumoniae* isolates. Among the 35 *P. aeruginosa* isolates tested in this study, the majority (27 isolates) exhibited an MIC = 8 μg/ml, indicating relatively good susceptibility to the antibiotic tested at this concentration (Fig. 2). However, 8 isolates of *P. aeruginosa* showed a higher MIC of 128 μg/ml, suggesting reduced susceptibility and potential resistance to this antibiotic. For *K. pneumoniae*, out of the 5 isolates examined, 3 isolates displayed an MIC of 8 μg/ml, similar to a significant (*p* < 0.05) proportion of *P. aeruginosa* isolates. One *K. pneumoniae* isolate demonstrated a higher MIC of 32 μg/ml, indicating decreased susceptibility, while another isolate showed a lower MIC of 2 μg/ml, suggesting higher susceptibility at a lower antibiotic concentration.

### Determination of *mcr-1* Gene

**Fig. 3:**
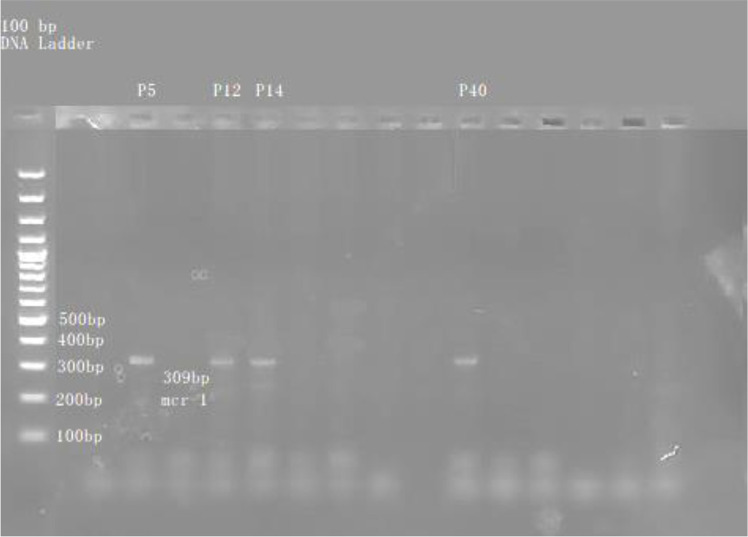
*mcr-1* gene detected in colistin-resistant isolates. Genetic detection of the *mcr-1* gene was conducted using conventional PCR across a total of 40 bacterial isolates. Among these six isolates, 15% were found positive for the *mcr-1* gene. (Note: The letter ‘P’ represents *P. aeruginosa*. In addition, P5, P12, P14, and P40 are the isolate numbers of *P. aeruginosa*).

In this study, genetic detection of the *mcr-1* gene was conducted using a conventional PCR across a total of 40 bacterial isolates. Among these six isolates, 15% were found positive for the *mcr-1* gene. Specifically, 4 out of 35 (11%) *P. aeruginosa* isolates and 2 out of 5 (40%) *K. pneumoniae* isolates tested positive for the presence of *mcr-1*. Notably, the remaining isolates did not carry the *mcr-1* gene (Table 2). Figure 3 represents the *mcr-1* gene detected in CR isolates.

## Discussion

In our study, we evaluated the antibiotic susceptibility profiles of *P. aeruginosa* and *K. pneumoniae* isolates, focusing on their MIC (Minimum Inhibitory Concentration) values against a specified antibiotic. Among the 35 *P. aeruginosa* isolates tested in this study, the majority (27 isolates) exhibited an MIC of 8 μg/ml, indicating a generally low level of resistance to this antibiotic at the tested concentration. However, 8 isolates demonstrated a higher 128 μg/ml, suggesting reduced susceptibility and potential resistance. For *K. pneumoniae*, our findings showed that three out of five isolates had an MIC of 8 μg/ml, aligning with the majority of *P. aeruginosa* isolates. Notably, one *K. pneumoniae* isolate had a significantly higher MIC of 32 μg/ml, indicating reduced susceptibility, while another isolate showed a lower MIC of 2 μg/ml, suggesting higher susceptibility. These results contribute to the broader understanding of antibiotic resistance patterns in these pathogens.

To provide a comprehensive analysis of antibiotic susceptibility, we included normal control isolates that exhibited typical antibiotic susceptibility profiles. These control isolates were sourced from non-infected patients and underwent the same MIC and antibiotic tests as the CR isolates. Our comparison showed that the normal control isolates of both *P. aeruginosa* and *K. pneumoniae* displayed significantly lower MIC values and higher susceptibility to colistin and other antibiotics tested, confirming their non-resistant status. For instance, the normal control isolates of *P. aeruginosa* had MIC values ranging from 0.5-2 μg/ml, while the *K. pneumoniae* control isolates had MIC values between 0.25 -1 μg/ml. This stark contrast in MIC values and susceptibility patterns highlights the elevated resistance observed in clinical isolates from infected wounds. The normal controls did not exhibit any resistance, emphasizing the concerning rise in resistance among the clinical isolates studied.

Comparing our findings with the recent studies, data from global surveillance and research publications highlight the ongoing challenges with antibiotic resistance in both *P. aeruginosa* and *K. pneumoniae*. Studies such as those by Yang *et al*. [26] have understood the persistence of multidrug resistance strains of *P. aeruginosa*, emphasizing the need for effective treatment strategies and surveillance measures. Moreover, reports by Peirano and Pitout [27] have documented the emergence of extended-spectrum beta-lactamases (ESBLs) and carbapenemase-producing *K. pneumoniae* isolates, which often exhibit high MIC values against various antibiotics, including those tested in our study. Additionally, surveillance data from the European Antimicrobial Resistance Surveillance Network (EARS-Net) have shown variable resistance rates across different European countries, reflecting the regional differences in antibiotic usage and resistance mechanisms.

According to our research, conventional PCR was used to detect the gene *mcr-1* in 15% of the bacterial strains we isolated - 11% of *P. aeruginosa* and 40% of *K. pneumoniae* strains proved to be carriers of this gene. The research findings here conform to earlier investigations, which give different *mcr-1* frequencies in patient specimens globally. For illustration, a report by Li *et al*. [28] found *mcr-1* in hospital-based *K. pneumoniae* strains in China, specifying its permanence as well as the likelihood that it may be spread in clinical settings. Mendes *et al*.[29] also found *mcr-1*-positive *P. aeruginosa* strains in South America, illustrating that its distribution is worldwide and important in various regions. In addition, data from an ECDC surveillance report shows that *mcr-1* is sporadically distributed in Europe, emphasizing the importance of continuous monitoring and infection control practices. Our conclusion serves as an addendum to these findings and underlines the need for a higher state of alertness in monitoring for new strains as well as ensuring that antibiotics are used properly so that CR caused by *mcr-1* cannot develop or spread.

The presence of the *mcr-1* gene is significant in that it encodes a phosphoethanolamine transferase enzyme, which modifies the lipid A component of the bacterial outer membrane, thereby reducing the binding affinity of colistin and leading to resistance [30, 31]. The existence of *mcr-1* in our isolates underscores the alarming capacity of these bacteria to develop resistance mechanisms that compromise last-resort antibiotics like colistin [32]. The high MIC values observed in isolates without the *mcr-1* gene suggest that other resistance mechanisms are at play [33]. These mechanisms may include chromosomal mutations leading to alterations in the target site of colistin efflux pump overexpression, or the presence of other resistance genes, such as *pmrA* and *pmrB* that regulate lipid A modification [34]. The diversity of resistance mechanisms highlights the complexity of antibiotic resistance and the necessity for comprehensive surveillance and tailored therapeutic strategies to manage infection caused by multidrug-resistant organisms.

### Study Area and Ethical Approval

The research was conducted between August 2023 and October 2023 at the Microbiology Laboratories of COMSATS University Islamabad (CUI), Lahore Campus, and the University of Central Punjab (UCP), Lahore, Pakistan. The Institutional Ethical Review Board (IREB) of UCP granted ethical approval with reference number FOST/DERC/2023/02. Prior to obtaining written informed permission from the patient/attendant, the patients were provided with comprehensive information about the nature and objective of the research. Due to the absence of further specimens and the lack of personal information received from patients, informed permission was not necessary for this investigation. The techniques were carried out in complete adherence to the criteria of the World Medical Association Declaration of Helsinki about the ethics for conducting medical research involving human subjects and identifiable human material/data.

## Figures and Tables

**Table 1:** The antibiotic susceptibility and resistance profile of carbapenem-resistant bacteria was analyzed in a sample size of 40.

Antibiotics	*Pseudomonas aeruginosa* (*n* = 35)		*Klebsiella pneumoniae* (*n* = 5)	
	S (%)	R (%)	S (%)	R (%)
Amikacin	5 (14)	30 (86)	1(20)	4 (80)
Ciprofloxacin	5 (14)	30 (86)	1(20)	4 (80)
Imipenem	1 (2)	34 (97)	0 (0)	5 (100)
Piperacillin/tazobactum	8 (23)	27 (77)	1(20)	4 (80)
Cefepime	1 (2)	34 (97)	0 (0)	5 (100)
Cotrimoxazole	2 (6)	33(94)	0 (0)	5 (100)
Ceftriaxone	0 (0)	35 (100)	0 (0)	5 (100)

**Table 2:** Detection of the *mcr-1* gene by PCR in bacterial isolates.

Strains	Total No. of Isolates	Mcr-1 Gene	No. of Isolates
*P. aeruginosa*	35	+	4
*K. pneumoniae*	5	+	2
